# High Temperature Resistant Separator of PVDF-HFP/DBP/C-TiO_2_ for Lithium-Ion Batteries

**DOI:** 10.3390/ma12172813

**Published:** 2019-09-02

**Authors:** Haijuan Li, Ling Li, Shuaizhi Zheng, Xinming Wang, Zengsheng Ma

**Affiliations:** National−Provincial Laboratory of Special Function Thin Film Materials and School of Materials Science and Engineering Xiangtan University, Hunan 411105, China

**Keywords:** TiO_2_ nanofiber, PVDF-HFP, composite separator, phase inversion, thermal shrinkage, ionic conductivity

## Abstract

To improve the thermal shrinkage and ionic conductivity of the separator for lithium-ion batteries, adding carboxylic titanium dioxide nanofiber materials into the matrix is proposed as an effective strategy. In this regard, a poly(vinylidene fluoride-hexafluoro propylene)/dibutyl phthalate/carboxylic titanium dioxide (PVDF-HFP/DBP/C-TiO_2_) composite separator is prepared with the phase inversion method. When the content of TiO_2_ nanofibers reaches 5%, the electrochemical performance of the battery and ion conductivity of the separator are optimal. The PVDF-HFP/DBP/C-TiO_2_ (5%) composite separator shows about 55.5% of porosity and 277.9% of electrolyte uptake. The PVDF-HFP/DBP/C-TiO_2_ (5%) composite separator has a superior ionic conductivity of 1.26 × 10 ^−3^ S cm^−1^ and lower interface impedance at room temperature, which brings about better cycle and rate performance. In addition, the cell assembled with a PVDF-HFP/DBP/C-TiO_2_ separator can be charged or discharged normally and has an outstanding discharge capacity of about 150 mAh g^−1^ at 110 °C. The battery assembled with the PVDF-HFP/DBP/C-TiO_2_ composite separator exhibits excellent electrochemical performance under high and room temperature environments.

## 1. Introduction

Lithium-ion battery (LIB) is the most promising power source for electronic devices because of its potential to be lightweight, high energy storage capability, long cycle life and pollution-free [1,2,3]. However, because of the frequent mobile phone explosions and computer burning incidents recently, the safety problem of lithium-ion batteries has attracted widespread attention, which severely hinders the application of lithium-ion batteries in daily life [4,5]. 

A complete lithium-ion battery is composed of an anode, an electrolyte, a separator, and a cathode. Among them, the separator plays a crucial role in the lithium-ion battery. The separator has a great electrical insulation performance to prevent internal short-circuiting and is the medium for lithium-ion transport [6]. Polyethylene (PE) and polypropylene (PP) are the widely used commercialized separators in lithium-ion batteries at present, owing to their excellent mechanical strength and chemical stability [7,8]. However, PE and PP materials have the disadvantages of low ion conductivity and poor compatibility with the electrolyte. Moreover, PE and PP separators are especially prone to heat shrinkage at high temperatures, which has raised serious internal short-circuiting and safety problems [9]. To overcome these drawbacks, Fu et al. [10] and Yoo et al. [11] proposed coating SiO_2_ nanoparticles on the surface of a commercial separator to enhance its thermal stability. Currently, the commonly used inorganic ceramic nanoparticles for coating include TiO_2_ [12], SiO_2_ [7,13], ZrO_2_ [14], Al(OH)_3_ [8] and Al_2_O_3_ [15,16]. Although the ceramic-coating on PE or PP separators can enhance heat resistance, the ionic conductivity and wettability of the separators are poor. In order to obtain a separator with a great thermal stability and electrochemical performance, some researchers propose to use poly(vinylidene fluoride-hexafluoro propylene) (PVDF-HFP) that has good compatibility with electrolyte as the matrix material. For example, Bottino et al. [17] and Pu et al. [18] studied a pure PVDF-HFP microporous separator prepared by the phase inversion method. On the basis of these works, many other researchers began to use the PVDF-HFP composite separator to improve the various properties of the separator. 

Recent research has focused on adding inorganic ceramic nanoparticles to the PVDF-HFP matrix, such as TiO_2_ [19,20], Al_2_O_3_ [16,21,22] and SiO_2_ [23], which increase the porosity of the composite separator because of the incompatibility of the polymer and inorganic ceramic nanoparticles. Kim et al. [24] had prepared a PVDF-HFP/TiO_2_ nanoparticles composite separator with superior electrochemical stability by the phase inversion method, but it had low ionic conductivity and non-uniform particle distribution. In addition, the thermal stability of those PVDF-HFP based composite separators should be further improved towards application under high temperatures. For example, the PP nonwoven/PVDF-HFP/fluorinated SiO_2_ nanoparticles composite separator [25] has a shrinkage of 17.5% at 150 °C after 0.5 h treatment, the Al_2_O_3_/PVDF-HFP based ceramic composite separator [15] and PP nonwoven/PVDF-HFP/PMMA blending-type composite separator [26] have a shrinkage of 59% and 19% after heat treatment at 140 °C for 0.5 h, respectively. In order to overcome the weakness of the particles’ aggregate phenomenon, multidimensional nanomaterials are applied, and they are attracting increasing attention [20,22,27]. For instance, one-dimensional TiO_2_ nanowire [20], polymer nanofiber [28] and two-dimensional clay nanosheets [9] are adopted. Moreover, the composite separators have high heat resistance, porosity, and high ionic conductivity. Nevertheless, the mechanical properties of the porous composite membranes are relatively lower, which may lead to the deformation or fracture of the separator and subsequently cause a short circuit in the battery. Hence, to improve the mechanical properties of the separator, a small amount of plasticizer can be added to ensure the stable mechanical properties [29]. 

Herein, the C-TiO_2_ nanofibers and dibutyl phthalate (DBP) were dispersed in PVDF-HFP solution using 1-methyl-2-pyrrolidone (NMP) as the solvent. The C-TiO_2_ nanofibers can improve the distribution through electrostatic repulsion of carboxyl groups from citric acid [22], which is profitable for enhancing the stability of the separator. Moreover, the addition of C-TiO_2_ nanofibers increases the porosity, the electrolyte uptake and the resultant ionic conductivity of the PVDF-HFP separator. Importantly, the high temperature resistant TiO_2_ nanofibers are also advantageous to suppress the thermal shrinkage. Meanwhile, DBP is added as the plasticizer for improving the mechanical properties. Generally, the 3D porous structure of the PVDF-HFP/DBP/C-TiO_2_ separator exhibits excellent thermal stability; high electrolyte uptake; and good wettability and electrochemical performances.

## 2. Materials and Methods 

### 2.1. Preparation of TiO_2_ Nanofibers

The TiO_2_ nanofibers were synthesized by the electrospinning method [30]. The mixed solution of tetrabutyl titanate (C_16_H_36_O_4_Ti) (Hangzhou Bangyi chemical Co., Ltd., Hangzhou, China), poly vinylpyrrolidone (PVP) (Aladdin, Shanghai, China), acetic acid (Xilong chemical Co., Ltd., Shantou, China) and ethanol (Tianjin Damao chemical reagent factory, Tianjin, China) was served as the precursor solution for TiO_2_ nanofibers. The TiO_2_ nanofibers were prepared at a high voltage (18–20 kV) and a feed rate of 1mL h^−1^. The samples were dried at 80 °C for 24 h followed by the calcination at 550 °C for 3 h in air to remove PVP.

The carboxylic-TiO_2_ (C-TiO_2_) nanofibers were synthesized by grinding the TiO_2_ nanofibers and citric acid monohydrate (Tianjin Hengxing chemical reagent manufacturing Co., Ltd., Tianjin, China) with a 1:0.5 weight ratio, then the mixed powder was dispersed in 30 mL deionized water and was stirred for 6 h. The resulted C-TiO_2_ nanofibers were centrifuged, washed with deionized water three times, and dried in the oven (Tianjin Taisite instrument Co., Ltd., Tianjin, China) at 80 °C for 12 h. Finally, the different contents of C-TiO_2_ nanofibers (0, 5, 10, and 15 wt%) were mixed with NMP solvent to make colloidal TiO_2_.

### 2.2. Preparation of Composite Separator

The precursor solution of the separator was prepared by dissolving 0.5 g PVDF-HFP powders in 5 mL NMP at 40 °C. When the mix solution became transparent, 300–350 μL of DBP was dropped into the transparent solution under constant stirring. Then, different contents of C-TiO_2_ were added into the solution and the mixture was kept stirring for 12 h to form a uniform solution. The PVDF-HFP/DBP/C-TiO_2_ solution was casted on a smooth glass plate by using a scraper. The glass plate was immersed in deionized water for 12 h to achieve a phase inversion process and extraction process of NMP. The composite separator was dried at 60 °C for 12 h.

### 2.3. Characterization

The morphology of the separators was tested by scanning electron microscopy (SEM, SU8010, Hitachi, Tokyo Japan) at an acceleration voltage of 15 kV. Fourier transform infrared spectroscopy (FTIR, Nicolet 6700, Shanghai, China) and X-ray diffraction (XRD, Rint2000, Rigaku Corporation, Tokyo, Japan) were applied to determine functional bond and crystallinity structure, respectively. Thermal stability of the separators was tested in a high and low temperature test chamber (HJ964641, Wuxi Huanshitong test equipment Co., Ltd., Wuxi, China). The thermal shrinkage ratio was calculated according to:

(1) $$T\left(\%\right)=\left(L_{0}-L\right)/L_{0}\times 100\%$$

here *L*_0_ and *L* are the diameter lengths of the separators before and after thermal treatment at various temperatures for 0.5 h, respectively. Electrolyte uptake (EU) of the composite separators was calculated as follows:

(2) EU(%)=(M−M0)/M0×100%

where *M*_0_ and *M* are the quality before and after immersion in electrolyte for 2 h, respectively. The porosity (*P*) of the separators determined by using the weight method was calculated based on the following Equation (3):

(3) $$P(\%)=\frac{\left(Mn-M0\right)/\rho n}{S\times d}\times 100\%$$

where *P* is the porosity of the separators, *M*_0_ and *M*_n_ are the initial mass of the separator and the mass after immersing in n-butanol for 2 h, respectively. *ρ*_n_ represents the density of the n-butanol, *S* is surface area of the separators and *d* is the thickness of the separators. The ionic conductivity was calculated with the following equation:

(4) σ=L/(Rb×As)

where σ is the ionic conductivity, *L* represents the thickness of the separators, *R*_b_ is the bulk resistance, *A*_s_ is the effective area of the separators disk. The ion conductivity measurements were carried out in the frequency range of 0.1 Hz to 100 kHz with an amplitude of 5 mV using electrochemical workstation (CHI660E, Xi’an, China). 

To measure the battery electrochemical performances, LiFePO_4_ and Li were used as the cathode and anode materials in coin batteries (CR2025), respectively. The LiFePO_4_ cathode was prepared by blending 70 wt% LiFePO_4_, 10 wt% PVDF powders and 20 wt% carbon black. The liquid electrolyte solution consists of 1 M LiPF_6_ in ethylene carbonate (EC), dimethyl carbonate (DMC) and ethyl-methyl carbonate (EMC) (1:1:1). The SS (stainless steel)/separator/SS and LiFePO_4_/separator/Li coin batteries were assembled in a glovebox (Mikrouna, Shanghai, China) filled with high purity argon gas. The charge-discharge performance of lithium-ion battery was tested at a rate of 0.5 C from 2.5 V to 3.6 V. The rate performance was measured at 0.5 C, 1 C, 2 C, and 3 C, respectively (10 cycles at each rate). The electrochemical impedance spectroscopy and ion conductivity were performed by electrochemical workstation at 0.1 Hz–100 KHz. The redox peak can be obtained by cyclic voltammetry at a scan rate of 1 mV in the range of 2.5 V to 4.5 V.

## 3. Results and Discussion

**Figure 1:**
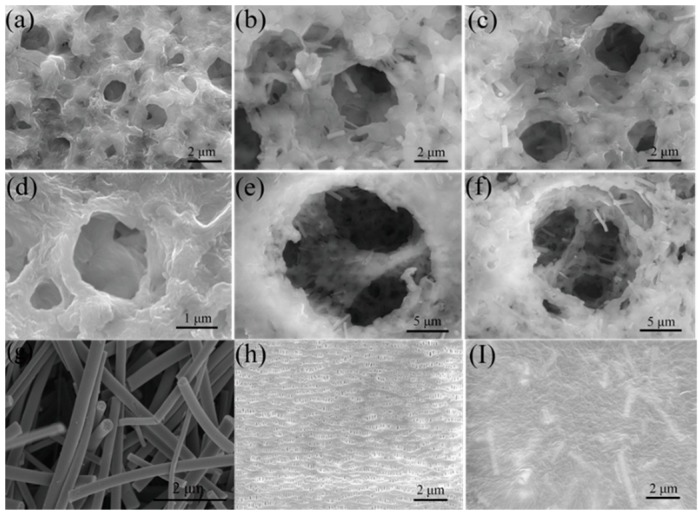
The SEM images of (**a**) and (**d**) pure PVDF-HFP separator, (**b**) and (**e**) PVDF-HFP/DBP/C-TiO_2_ (5%) separator, (**c**) and (**f**) PVDF-HFP/DBP/C-TiO_2_ (10%) separator, (**g**) TiO_2_ nanofibers, (**h**) PP separator, (**i**) PVDF-HFP/DBP/C-TiO_2_ (5%) separator after heating at 150 °C for 0.5 h.

A uniform PVDF-HFP/DBP/C-TiO_2_ separator has been prepared, as shown in Figure 1. The average diameter of the doped TiO_2_ nanofibers is about 0.5 μm. Figure 1a,d displays the morphology of the pure PVDF-HFP separator. The pore size of the separator is about 1–3 μm and the pore distribution is uniform. The wall of the PVDF-HFP separator is smooth and the holes overlap and interconnect with each other. Indeed, this pore structure can improve the storage ability for electrolyte and prevent lithium dendrite from penetrating the separator [31]. As presented in Figure 1b,c,e,f, the C-TiO_2_ nanofibers are uniformly distributed in PVDF-HFP/DBP/C-TiO_2_ composite separators, and the pore size is 2–5 μm. From the enlarged drawing of the porous composite separator (Figure 1e,f), the PVDF-HFP/DBP/C-TiO_2_ composite separators still have interconnected 3D pore structures and many small voids appear on the walls of the pores, which make PVDF-HFP/DBP/C-TiO_2_ composite separators possess higher porosity and electrolyte uptake rate than PP and PVDF-HFP separators. The 3D porous structure is beneficial for the electrolyte uptake, wettability, and is helpful to suppress the growth of Li dendrites [31]. In general, the pore structure of the PVDF-HFP/DBP/C-TiO_2_ separator is more complex and larger than the PP separator. In addition, Figure 1i shows the surface morphology of PVDF-HFP/DBP/C-TiO_2_ (5%) composite separators after heat treatment at 150 °C. It can be found that the pore structure of PVDF-HFP/DBP/C-TiO_2_ (5%) composite separators disappeared and the surface of the composite separators became flat. The disappeared pores impede the diffusion of Li^+^, which makes the battery unable to be charged and discharged normally. This result indicates that the composite separators offer a thermal shutdown property.

**Figure 2:**
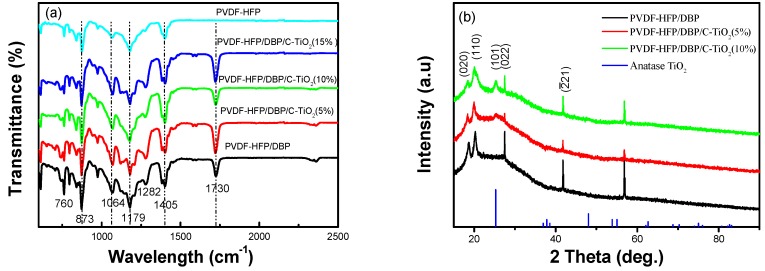
(**a**) FTIR spectra of pure PVDF-HFP, PVDF-HFP/DBP and PVDF-HFP/DBP/C-TiO_2_ (5%, 10%, 15%) composite separators, (**b**) XRD spectra of PVDF-HFP/DBP, PVDF-HFP/DBP/C-TiO_2_ (5%, 10%) composite separators.

To determine the functional group and crystal structure of the composite separators, Fourier transform infrared spectroscopy (FTIR) and X-ray diffraction (XRD) spectra are measured and exhibited in Figure 2. The absorption peaks at 1405 and 760 cm^−1^ (Figure 2a) can be attributed to the bending vibration absorption peaks of CH_2_. The asymmetrical and symmetrical stretching vibrations of CF_2_ are present at 1179 and 1282 cm^−1^ [22]. Meanwhile, the peak for β-phase of PVDF-HFP appears at 873 cm^−1^ [32,33]. In general, these are characteristic peaks of PVDF-HFP, and they are consistent with the results of the following XRD spectrum analysis. The peaks at 18.4°, 20.06°, 27.04° and 41.8° are consistent with the (020), (110), (022) and ($\bar{2}21$) crystalline planes of PVDF-HFP in Figure 2b. Besides, adding TiO_2_ nanofibers into PVDF-HFP leads to a small decrease in the peak intensities. Probably, it is resulted from the increased amorphous structure of the PVDF-HFP separator, which is beneficial for a lithium ion battery [8,34]. In addition, the diffraction peak of TiO_2_ appears at 25.3° is consistent with the (101) crystalline plane, and the peak intensity gradually increases with the concentration of TiO_2_ nanofibers. The C=O and C–O stretching absorption peaks of DBP and citric acid (C_6_H_8_O_7_) appear at 1730 and 1064 cm^−1^ [35]. Combining these results, it is indicated that the composite separators are composed of PVDF-HFP, DBP and TiO_2_ nanofibers.

**Figure 3:**
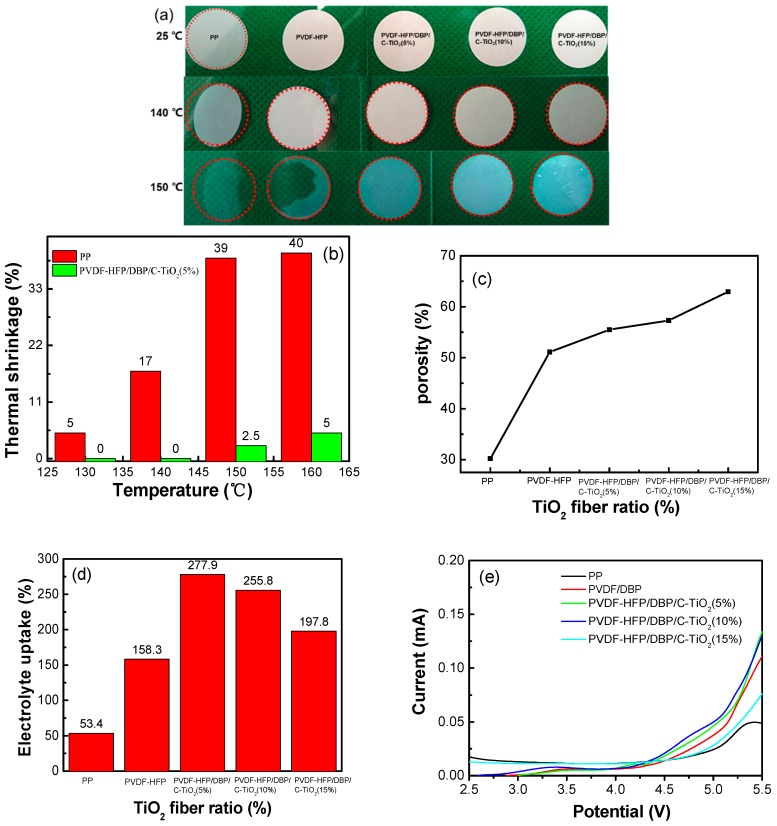
(**a**) Photographs of the separators after heat treatment in an oven at 25 °C, 140 °C and 150 °C for 0.5 h, (**b**) thermal shrinkage rates of separators at different temperatures, (**c**,**d**) porosity and electrolyte uptake of different composite separators, (**e**) linear sweep voltammetry (LSV) of PP, PVDF-HFP/DBP and PVDF-HFP/DBP/C-TiO_2_ composite separators.

Thermal stability of the composite separators plays an important role for the safety of lithium ion batteries under some extreme conditions. Figure 3a shows the thermal shrinkage photographs of the separators before and after thermal treatment in an oven at 25 °C, 140 °C and 150 °C for 0.5 h. Generally, the commercial separator has an obvious thermal shrinkage and transparency phenomenon above 140 °C. For example, the thermal shrinkage of the PP separator is about 40% at 150 °C, while the PVDF-HFP/DBP/C-TiO_2_ (5%) separator only shows a thermal shrinkage of 2.5%. The thermal shrinkage of the PVDF-HFP/DBP/C-TiO_2_ (10%) separator and the PVDF-HFP/DBP/C-TiO_2_ (15%) separator are close to that of the PVDF-HFP/DBP/C-TiO_2_ (5%) separator. The color of the separator changes from white to translucent after heat treatment due to the polymer melting at high temperature. As shown in Figure 1i, the surface of the heated separator is smoother and the microporous structure of the composite separator disappears, which can be attributed to the melting of PVDF-HFP.

Figure 3c is the porosity statistics chart of the composite separators. It clearly shows that the porosity of PVDF-HFP/DBP/C-TiO_2_ composite separators gradually increases along with the growing content of TiO_2_ nanofibers. The porosity of PVDF-HFP/DBP/C-TiO_2_ composite separators far exceeds that of the PP separators (30.2%), which is related to the 3D porous structure. For instance, the PVDF-HFP/DBP/C-TiO_2_ (5%) composite separator shows about 55.5% of porosity. In addition, the electrolyte uptake of PVDF-HFP/DBP/C-TiO_2_ composite separators is better than that of the commercial PP (53%) and the PVDF-HFP/DBP composite separators (158.3%). The high porosity is beneficial to improve the electrolyte uptake and wettability of the separator. The optimum electrolyte uptake of PVDF-HFP/DBP/C-TiO_2_ (5%) composite separator achieves 277.9% However, the electrolyte uptake has a descending trend, when the amount of TiO_2_ nanofibers is excessive. The electrolyte uptake of the PVDF-HFP/DBP/C-TiO_2_ (10%) and PVDF-HFP/DBP/C-TiO_2_ (15%) composite separators are about 255.8% and 197.8%, respectively. As shown in Figure 3e, the electrochemical working window is measured by linear sweep voltammetry. The anodic current starts to increase at 4.3 V for composite separators, indicating the decomposition of EC (ethylene carbonate) and DMC (dimethyl carbonate) [36]. Therefore, the composite separators could be used as the separators for lithium batteries, since the charging-discharging voltage of lithium-ion batteries is below 4.2 V.

**Figure 4:**
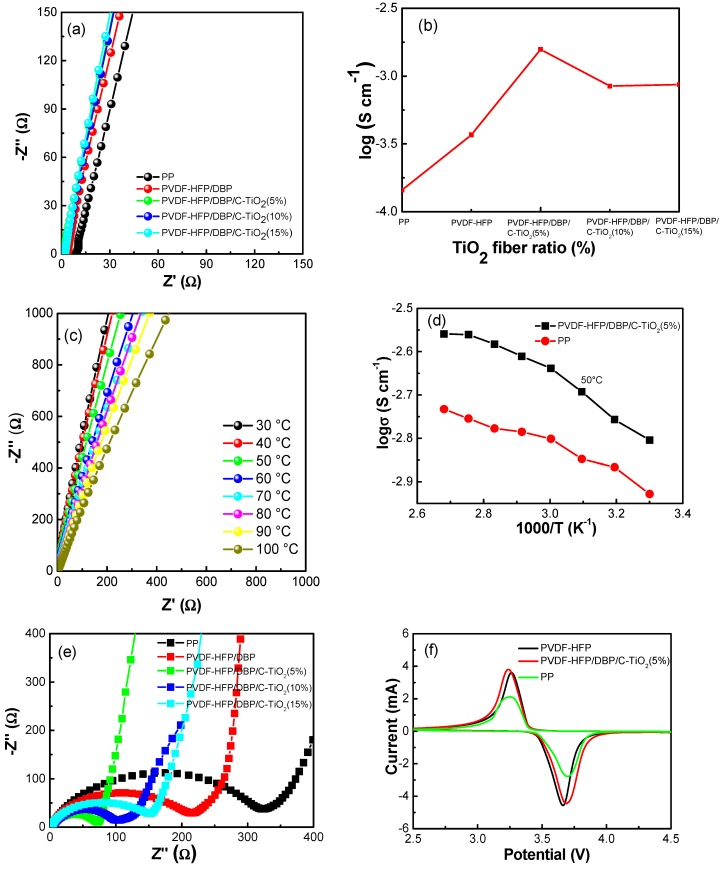
(**a**) AC impedance spectra, (**b**) ionic conductivity of stainless steel/separator/stainless steel cells assembled with different separators, (**c**) AC impedance spectra of PVDF-HFP/DBP/C-TiO_2_ (5%) composite separator at different temperatures, (**d**) ionic conductivity of PP and PVDF-HFP/DBP/C-TiO_2_ (5%) composite separators at different temperatures, (**e**) electrochemical impedance spectra, and (**f**) cyclic voltammogram curves of LiFePO_4_/separator/Li cells with the PP, PVDF-HFP/DBP, and PVDF-HFP/DBP/TiO_2_ (5%) composite separators.

Figure 4a,b presents the bulk resistance and ionic conductivity of different composite separators at room temperature. The bulk resistance of PVDF-HFP/DBP/C-TiO_2_ separators is smaller than those of the pure PVDF-HFP and PP separators. According to Equation (4), the ionic conductivity of PVDF-HFP/DBP/C-TiO_2_ (5%) separators is 1.26 × 10^−3^ S cm^−1^ at 25 °C, while for the PP, PVDF-HFP/DBP, PVDF-HFP/DBP/C-TiO_2_ (10%) and the PVDF-HFP/DBP/C-TiO_2_ (15%) separators, the ionic conductivities are 1.51 × 10^−4^ S cm^−1^, 3.69 × 10^−4^ S cm^−1^, 8.45 × 10^−4^ S cm^−1^ and 8.66 × 10^−4^ S cm^−1^, respectively. The higher ionic conductivity of the composite separators is mainly due to the 3D pore structure with larger porosity engendered by adding C-TiO_2_ nanofibers. This interconnected 3D pore structure provides a channel for Li^+^ diffusion, which makes it more easily for Li^+^ to cross the separator. Furthermore, the ionic conductivity of the composite separators is also tested under different temperatures (Figure 4c,d). The bulk resistance decreases, and ionic conductivity increases gradually with the increase of temperature. The reason is that higher temperature can improve the migration rate of Li^+^ in the composite separators [22,37]. When the temperature exceeds 50 °C, the ionic conductivity of PVDF-HFP/DBP/C-TiO_2_ (5%) composite separator has a higher growth rate than that of the PP separator. Therefore, the PVDF-HFP/DBP/C-TiO_2_ composite separator is more suitable for battery application at a high temperature.

To further understand the role of the additives in PVDF-HFP composite separators, various electrochemical performances were measured. As shown in Figure 4e, the charge transfer resistance of PVDF-HFP and PP separators are 217 and 325 Ω, respectively. Notably, the electrochemical impedance of PVDF-HFP/DBP/C-TiO_2_ separators owns a lower charge transfer resistance than those of the pure PVDF-HFP and PP separators. The PVDF-HFP/DBP/C-TiO_2_ (5%) composite separator has the lowest charge transfer resistance about 75 Ω among the composite separators. It can be attributed to the higher wettability and porosity of the separator, which reduce the impedance of the cell [38]. The cyclic voltammetry curve is taken at a scan rate of 1 mV s^−1^ between 2.5 and 4.5 V. The reduction and oxidation peaks are 3.7 and 3.3 V, respectively. The redox couple corresponds to the deintercalation and intercalation of lithium ions from LiFePO_4_. 

**Figure 5:**
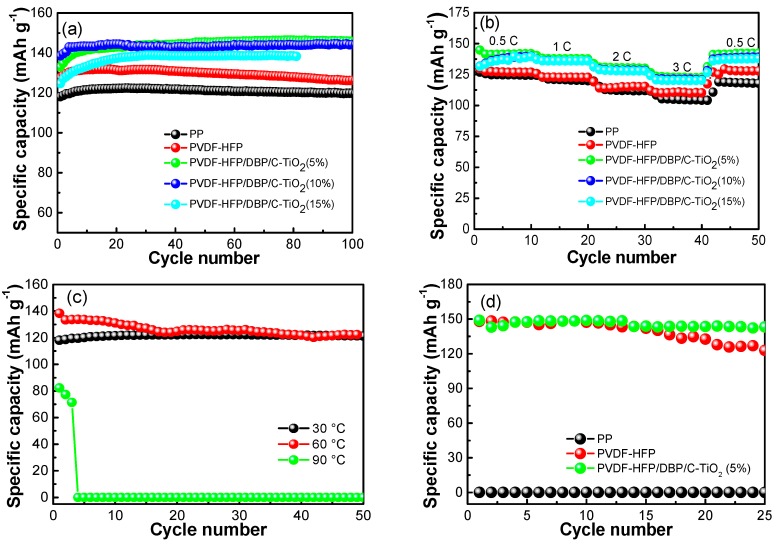
(**a**,**b**) the cycle and rate performance of cells with different separators, (**c**) cycle performance of battery assembled with PP separator at 30, 60 and 90 °C, (**d**) cycle performance of cells assembled with PP, PVDF-HFP/DBP and PVDF-HFP/DBP/C-TiO_2_ (5%) composite separators at 110 °C.

In comparison, a series of electrochemical performances of the batteries with different separators were measured. As demonstrated in Figure 5a, coin cells with PP membranes have a lower discharge capacity about 125 mAh g^−1^ than composite separators at 30 °C. The batteries assembled with PVDF-HFP/DBP/C-TiO_2_ (5%, 10%) composite separators have the discharge capacity of above 145 mAh g^−1^. Although the batteries with pure PVDF-HFP membranes have higher capacity than batteries with PP, their electrochemical stability and capacity are inferior to cells assembled with PVDF-HFP/DBP/C-TiO_2_ composite separators. Reasonably, the addition of TiO_2_ nanofibers can ameliorate the shortcoming of a PVDF-HFP separator. However, the electrochemical performance of batteries with PVDF-HFP/DBP/C-TiO_2_ separators decays when the doping amount of TiO_2_ nanofibers is in excess. On the other hand, the rate performance of batteries observed from Figure 5b, shows that cells assembled with PVDF-HFP/DBP/C-TiO_2_ composite separators have higher discharge capabilities than those assembled with PP and pure PVDF-HFP separators at different discharge rates. The specific capacity of batteries with composite separators are about 140 and 130 mAh g^−1^ at 0.5 and 3 C, respectively, while batteries with the pure PVDF-HFP and PP separators deliver the capacities of 125 and 105 mAh g^−1^ at 0.5 and 3 C, respectively. Moreover, it can be found that batteries assembled with PVDF-HFP/DBP/C-TiO_2_ composite separators have greater rate performance at 30 °C. The improved cycle and rate performances are mainly attributed to the higher ionic conductivity and lower interfacial resistance. In brief, these results illustrate that the batteries with PVDF-HFP/DBP/C-TiO_2_ composite separators have better electrochemical stability, ionic conductivity and electrochemical performances.

In order to compare the heat-resistant temperature of the battery equipped with PP separator, the electrochemical performances of cells at different temperatures were measured and the results were shown in Figure 5c. These cells have a stable charge-discharge specific capacity at 30 °C, while specific capacity of a cell assembled with PP is slightly decayed with cycle numbers at 60 °C. When temperature is 90 °C, a short circuit occurs after only three cycles. In addition, Figure 5d reveals the discharge capacity of cells with different separators at 110 °C. Because the electrolyte is prone to thermal decomposition at 110 °C, the batteries are set to run for 25 cycles. The battery assembled with PP cannot be charged and discharged normally. It could be caused by thermal shrinkage of the micropores in the PP membranes with low dimensional stability at 110 °C [28]. In contrast, at the same high temperature, the batteries assembled with PVDF-HFP/DBP/C-TiO_2_ composite separators demonstrate an excellent high temperature resistance and an outstanding discharge capacity about 150 mAh g^−1^. Despite that battery assembled with the pure PVDF-HFP composite separator can be discharged normally, the battery has a low electrochemical stability. Therefore, the discharge capacity of the battery has a decreasing trend with the number of cycles. In sum, the above results clearly demonstrate that batteries assembled with PVDF-HFP/DBP/C-TiO_2_ composite separators have superior thermal stability and electrochemical performances at 110 °C.

## 4. Conclusions

Thermally stable PVDF-HFP/DBP/C-TiO_2_ separators are prepared by a phase inversion method. The 3D microporous structure of the composite separators is crucial to ameliorate the interfacial resistance and ionic conductivity. Moreover, the TiO_2_ nanofibers can improve the thermal stability of the separator. The PVDF-HFP/DBP/C-TiO_2_ (5%) composite separator has an electrolyte uptake of 278% and porosity of 63%, and the ionic conductivity of the PVDF-HFP/DBP/C-TiO_2_ (5%) electrolyte system can reach 1.26 × 10^−3^ S cm^−1^ at 25 °C. Meanwhile, the battery assembled with PVDF-HFP/DBP/C-TiO_2_ (5%) composite separator exhibits better cycling and rate performance than those assembled with PP and pure PVDF-HFP separators. The PVDF-HFP/DBP/C-TiO_2_ separators possess thermal shrinkage at high temperatures, and the cell assembled with this composite separator also shows superior electrochemical performance and high-temperature resistance. The battery assembled with the PVDF-HFP/DBP/C-TiO_2_ separator has a discharge capacity of 150 mAh g^−1^ at 110 °C. The overall results illustrate that the PVDF-HFP/DBP/C-TiO_2_ separator has promising potential for LIBs applications under a high temperature environment.
